# Development and application of a 1K functional liquid chip for lactation performance in Bactrian camels

**DOI:** 10.3389/fvets.2024.1359923

**Published:** 2024-07-02

**Authors:** Lili Guo, Lema Dao, Bin Liu, Jingyu Wang, Zaixia Liu, Fengying Ma, Bielige Morigen, Chencheng Chang, Yinbatu Bai, Yaqiang Guo, Caixia Shi, Junwei Cao, Wenguang Zhang

**Affiliations:** ^1^College of Life Science, Inner Mongolia Agricultural University, Hohhot, China; ^2^Inner Mongolia Engineering Research Center of Genomic Big Data for Agriculture, Inner Mongolia Agricultural University, Hohhot, China; ^3^Inner Mongolia Autonomous Region Key Laboratory of Biomanufacturing, Hohhot, China; ^4^Bactrian Camel Institute of Alsha, Bayanhot, China; ^5^Inner Mongolia Bionew Technology Co., Ltd., Hohhot, China; ^6^College of Animal Science, Inner Mongolia Agricultural University, Hohhot, China; ^7^Animal Disease Prevention and Control Center of Alsha, Bayanhot, China

**Keywords:** lactation performance, SNP markers, RNA-seq, liquid chip, Bactrian camel

## Abstract

**Introduction:**

The advancement of high-throughput, high-quality, flexible, and cost-effective genotyping platforms is crucial for the progress of dairy breeding in Bactrian camels. This study focuses on developing and evaluating a 1K functional liquid single nucleotide polymorphism (SNP) array specifically designed for milk performance in Bactrian camels.

**Methods:**

We utilized RNA sequencing data from 125 lactating camels to identify and select 1,002 loci associated with milk production traits for inclusion in the SNP array. The array’s performance was then assessed using 24 randomly selected camels. Additionally, the array was employed to genotype 398 individuals, which allowed for population validation to assess the polymorphism of SNP sites.

**Results:**

The SNP array demonstrated high overall SNP call rates (> 99%) and a remarkable 100% consistency in genotyping. Population validation results indicate that camels from six breeding areas in Northwest China share a similar genetic background regarding lactation functionality.

**Discussion:**

This study highlights the potential of the SNP array to accelerate the breeding process of lactating Bactrian camels and provides a robust technical foundation for improving lactation performance.

## 1 Introduction

The versatile applications of Bactrian camels, such as their provision of milk, meat, and down, in addition to their adaptability to various ecological conditions, render them a crucial species in both contemporary and conventional agricultural practices. Notably, their milk is not only a dietary staple but also renowned for its medicinal properties, making it a crucial resource for both nutritional and therapeutic purposes (1–3). However, the full potential of Bactrian camel milk production is yet to be harnessed, often limited by the lack of advanced breeding strategies that focus on lactation performance traits.

Single nucleotide polymorphism (SNP) chips are transforming animal breeding; low cost “assay-by-sequencing” methodologies and high quality reference genome sequences provide the opportunity for further significant improvement in both breeding and management (4). Functional SNPs are generally defined as SNPs from genome sequences that affect structure, expression, or function of a gene. SNPs, which were located within expressed genes, are especially important because they have the potential to change the function of a protein (5). RNA sequences are exclusively transcribed from exonic regions of the genome, making them ideal candidates for developing markers specific to these genic regions. Such markers are particularly valuable in domestic animal breeding. Several studies used RNA sequencing to identify markers in human (6) and animals (7–14). However, the genetic underpinnings of lactation traits in Bactrian camel remain insufficiently explored.

Single nucleotide polymorphism (SNP) arrays represent a high-quality and user-friendly platform for genotyping (15, 16). Utilizing a SNP array enables the simultaneous detection of tens of thousands of SNPs per sample, thereby facilitating high-throughput and efficient methodologies in genetic research and breeding programs. More recently, advances have led to the development of a liquid SNP chip panel, leveraging Genotyping By Targeted Sequencing (GBTS) technology (17). This innovation aims to further reduce costs and enhance the accuracy of genomic selection. When compared to traditional SNP chips that utilize magnetic beads, liquid-phase chips offer several advantages, including reduced cost and increased flexibility (17, 18).

However, despite the widespread application of various high-throughput methods in the genetic study of dairy cattle, poultry, and aquatic animals, their utilization remains limited in Bactrian camel research. Based on high-quality SNPs selected from transcribed regions, GBTS was utilized to develop a 1K functional SNP liquid array, named “CamelBell No. 1,” for Bactrian camels. The genotyping performance and prediction accuracy of the 1K SNP liquid array were validated. This array will become a valuable tool for enhancing Bactrian camel milk performance, attributable to its stable genotyping capabilities and its robust correlation with lactation traits in Bactrian camels.

## 2 Methods

### 2.1 Samples collection

Phenotypic data, along with milk and blood samples, were meticulously collected from 125 lactating Alxa Desert Bactrian camels located at the Alashan League, Inner Mongolia. All camels were in a consistent lactation period, with a parity ranging from 2 to 5, and were maintained in optimal body condition within a uniform environment. They were fed the same diet: dry clover supplemented with 2 kg of grain concentrate (68% corn +12% wheat bran +20% soybean cake after oil extraction) and 30 g of table salt for each animal daily. Blood and milk samples were collected from 32 and 83 Bactrian camels at approximately 30 and 270 days postpartum, respectively. Daily milk production was estimated based on the average milk yield over three consecutive days. For compositional analysis, 25 mL of milk was sampled to determine the concentrations of key constituents such as fat, protein, and lactose using mid-infrared spectrometry (MilkoScan Minor, Foss Analytics, Hillerød, Denmark). Additionally, 10 mL of whole blood was collected from the jugular vein of each camel for genetic analysis. Blood samples were treated with TRIzol reagent (TaKaRa, United States) and stored at −80°C until RNA extraction could be performed.

Genomic DNA was collected from 398 Bactrian camels used for chip genotyping. These camels were from six key breeding regions in China, which are major areas for Bactrian camel breeding. Specifically, samples were obtained from Subei County, Gansu (GSB; 7 individuals), Alxa Right Banner, Inner Mongolia (NAY; 82 individuals), Alxa Left Banner, Inner Mongolia (NAZ; 257 individuals), Sunit Right Banner, Inner Mongolia (NSNT; 22 individuals), Siziwang Banner, Inner Mongolia (NSZW; 10 individuals), and Urad Rear Banner, Inner Mongolia (NWLT; 20 individuals). The dataset comprises three breeds of Bactrian camels: Alxa Desert Camel (*n* = 343), Alxa Gobi Camel (*n* = 22), and Sunit Bactrian Camel (*n* = 33). Alxa Desert Camels were sampled from GSB, NAY, and NAZ, while Alxa Gobi Camels were exclusively sampled from NWLT, and Sunit Bactrian Camels were sampled from NSNT and NSZW. The methodologies used in this study were approved by the Institutional Animal Care and Use Committee of Inner Mongolia Agricultural University, Hohhot, China.

### 2.2 RNA extraction and sequencing

We isolated total RNA from 125 blood samples using the TRIzol reagent (Invitrogen, Carlsbad, CA, United States). The blood samples were homogenized in TRIzol reagent and chloroform, followed by precipitation using isopropanol. Total RNA from each sample was treated for genomic DNA contamination using the RNase-free DNase set (QIAGEN, Crawley, West Sussex, United Kingdom) and purified using the RNeasy mini kit according to the supplied guidelines (QIAGEN, Crawley, West Sussex, United Kingdom). RNA sample quality was assessed using the NanoPhotometer^®^ spectrophotometer (IMPLEN, CA, United States) and the Agilent Bioanalyzer 2100 system. RNA samples exhibited 28 S/18 S ratios ranging from 1.8 to 2.0 and RNA integrity number values between 8.0 and 10.0.

For mRNA cDNA library preparation, 1.0 μg of total RNA was utilized from each sample using the TruSeq RNA Library Preparation kit v2 (Illumina, San Diego, CA, United States). Poly A-containing mRNA was enriched from the total RNA using poly-T oligo attached beads and fragmented for first-strand cDNA synthesis, followed by second-strand synthesis. The ends were repaired, and 3′ end adenylation and adapter ligation were performed for each library. Subsequently, libraries were polymerase chain reaction (PCR) amplified, validated using the Bioanalyzer (Agilent Technologies Inc., Cedar Creek, TX, United States), and finally normalized and pooled. Clustering of the index-coded samples was conducted on a cBot Cluster Generation System using TruSeq PE Cluster Kit v3-cBot-HS (Illumina) according to the manufacturer’s instructions. Following cluster generation, the library preparations were sequenced on the Illumina NovaSeq 6000 high-throughput sequencing platform, generating 150 bp paired-end reads.

### 2.3 SNP detection and selection

**Figure 1:**
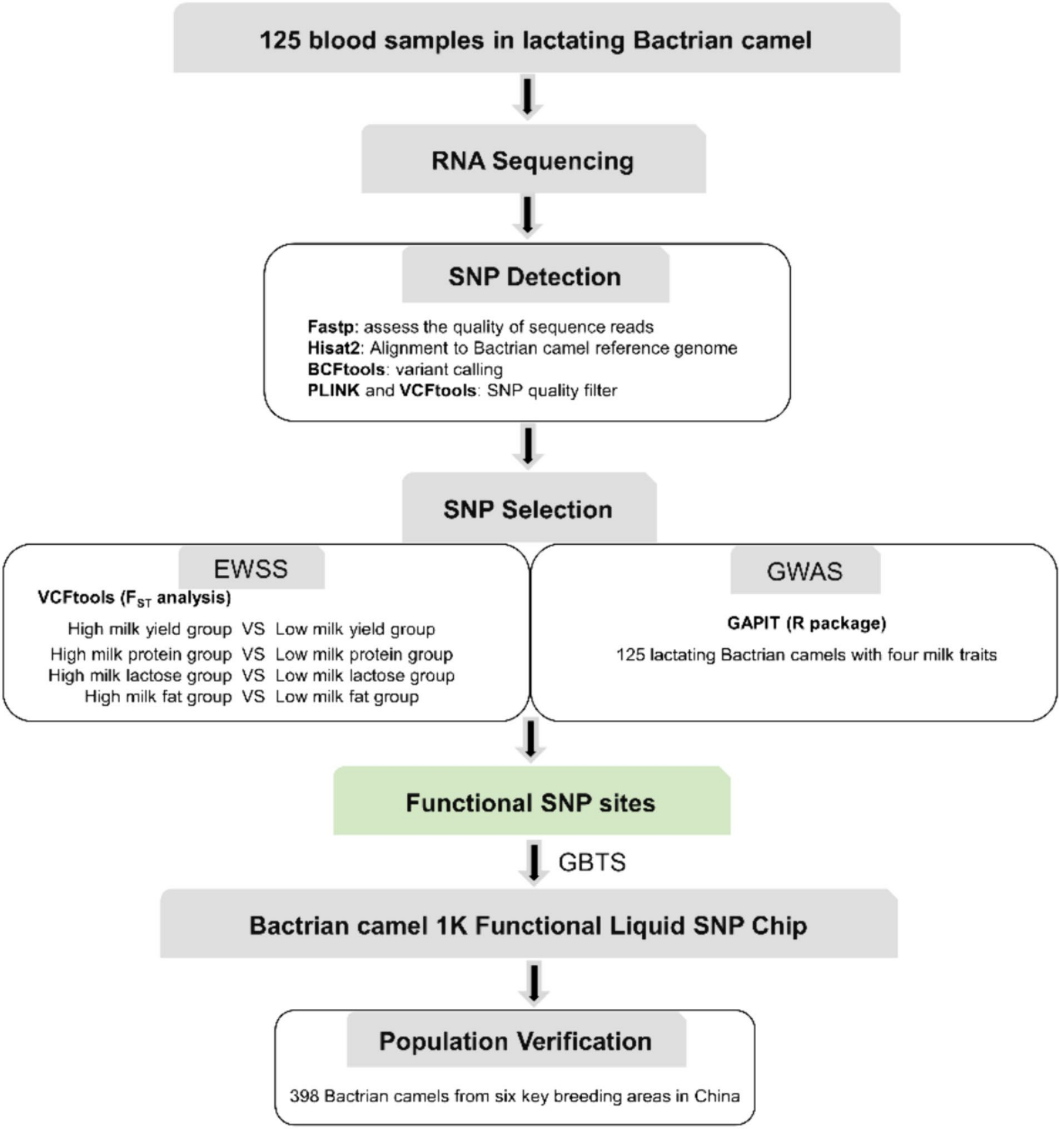
The workflow used for SNP chip design procedure.

The identification of SNPs was executed following the workflow depicted in Figure 1, with the comprehensive SNP calling pipeline script provided in Supplementary file S4. Fastp v0.23.4 (19) was employed to assess the quality of sequence reads, targeting the identification of sequencing read artifacts such as low-quality Phred scores, duplicated reads, uncalled bases (N sequences), and potential contamination. Subsequently, the filtered reads from each sample underwent individual alignment to the Bactrian camel reference genome (Ca_bactrianus_MBC_1.0) using Hisat2 v2.1.0 software (20). The aligned results were exclusively utilized for Single Nucleotide Polymorphism (SNP) calling, while insertions and deletions (indels) were excluded from the analysis due to challenges associated with accurate indel calling. Variant calling was performed for each read merging method using the “mpileup” and “call” commands from BCFtools v1.9–77-gd0cf724+ (21). Only those SNP variants were retained where the alternative allele manifested in all samples, accompanied by a Phred quality score of at least 25 and a minimum read depth of 10. We calculated the minor allele frequencies (MAFs) and missing rates using PLINK v1.90 (22), and the SNPs with MAF <0.05 were excluded. We tested the Hardy–Weinberg equilibrium (HWE) using VCFtools v0.1.11 (23) with the -hwe option and removed SNPs that severely departed from HWE (*p* < 0.01).

### 2.4 Development of the SNP panel

#### 2.4.1 SNP identification with camel milk traits

To identify SNPs crucial to Bactrian camel lactation and to develop functional chips essential for enhancing their breeding, we conducted Exon-Wide Selection Signature (EWSS) and Genome-Wide Association Study (GWAS) algorithm (24) analyses on carefully evaluated SNPs. Initially, EWSS is an SNP screening method based on transcription level by detecting the *F*_ST_ index of population differentiation. Thus, from 83 Bactrian camels at 270 days postpartum, we selected 30 individuals each, based on traits such as milk yield, milk protein, lactose, and milk fat, and conducted *F*_ST_ analysis on these extreme groups using VCFtools v0.1.11 (23). The single-locus *F*_ST_ for each SNP in transcriptomic regions was calculated, and those with an *F*_ST_ greater than 0.2 were retained. The Genome-Wide Association Study (GWAS) analysis was conducted utilizing the Genomic Association and Prediction Integrated Tool (GAPIT) within the R programming environment (25). The association analysis examined individual markers among 19,177 single nucleotide polymorphisms (SNPs) in relation to the best linear unbiased estimate (BLUE) value of each accession for each trait. The BLUE values provide unbiased estimates of phenotypic traits, incorporating fixed effects such as lactation stage and parity within the model. For population correction and stratification within the mixed linear model (MLM), both a kinship matrix and principal component analysis (PCA) were computed. The kinship matrix accounts for the genetic relatedness among individuals, while PCA addresses population structure, thereby mitigating the risk of false positives attributable to population stratification. *p*-values were subsequently adjusted at a 5% false discovery rate (FDR) to ascertain significant associations. To determine the relevance of the applied model for GWAS, quantile–quantile (QQ) plot was derived among the observed and expected log10(*p*) value. The workflow used for SNP chip design procedure is summarized in Figure 1.

#### 2.4.2 Variants annotation and enrichment analysis

Single nucleotide polymorphism (SNP) annotations, categorized by functional class (such as genic or intergenic), along with their genomic distributions, were performed using custom Perl scripts. The annotation process utilized the Generic Feature Format (GFF) file^1^ of the Bactrian camel genome reference (Ca_bactrianus_MBC_1.0). This file provided the necessary information to determine the genomic context of each SNP. A SNP was classified as genic if it was located within the start and end positions of an mRNA transcript, which includes coding sequences (CDS), 5′ untranslated regions (5′UTR), or 3′ untranslated regions (3′UTR). Conversely, SNPs that did not fall within these mRNA boundaries were designated as intergenic.

For the Gene Ontology (GO) and Kyoto Encyclopedia of Genes and Genomes (KEGG) enrichment analyses, we utilized the g:Profiler^2^ web interface (26). This platform facilitated the identification of significant biological processes, cellular components, and molecular functions (as classified by GO), as well as the pathways (as cataloged in KEGG) that are overrepresented in our gene set. To account for the issue of multiple comparisons, which can lead to false positives, we employed Bonferroni for multiple testing correction. Only results with an adjusted *p*-value below 0.05 were considered statistically significant.

#### 2.4.3 Design and synthesis of the liquid chip

The liquid chip utilizes GBTS technology (18, 27, 28), which operates on the principle of target capture through the complementary pairing of probes with the target sequences. In this study, we initially assessed and scored the selected candidate loci using the Compass probe design system (Compass Biotechnology, Guilin, China). The probe design considered the complexity and GC content of the sequences upstream and downstream of the target loci to ensure accuracy and efficiency. Priority was given to positioning the target loci in the middle of the probes, each designed to be 120 bp in length. After verification, these loci were submitted to Compass for probe synthesis, with each probe modified with a biotin group at the 5′ end to facilitate subsequent experimental steps.

#### 2.4.4 DNA extraction and library construction

DNA was extracted from the 398 samples using a magnetic bead method, known for its efficiency and ability to yield high-quality DNA. The extracted DNA was then fragmented to align with the PE150 sequencing strategy, targeting a main fragment size range of 200–300 bp. This fragmentation was followed by selection, end-repair, and A-tailing, preparing the DNA for sequencing library construction. The library construction process involved the ligation of sequencing adapters and PCR amplification to enrich the target fragments, ensuring comprehensive coverage. Subsequently, the libraries were quantified using the dsDNA HS Assay Kit for Qubit. Electrophoresis was employed to confirm that their main peak sizes fell within the 350–450 bp range, a step crucial for ensuring sequencing quality and accuracy.

For hybrid capture, pooled libraries, comprising the whole-genome DNA from each sample, were prepared, totaling 4 μg per hybrid capture library. These pooled libraries were then concentrated and subjected to probe hybridization, specifically targeting fragments from the whole-genome library. Following hybridization, excess probes, reagents, and other components were removed during the elution process. Post-hybridization, PCR amplification was performed to further enrich the target regions, culminating in the final library ready for sequencing. These libraries were quantified and subsequently sequenced using the MGI-T7 sequencer.

### 2.5 SNP array performance evaluation

To assess the stability and reliability of the “CamelBell No. 1” SNP chip, we performed genotyping on 24 lactating camels randomly selected from the 398 DNA samples. The sample set included three pairs of duplicate samples to evaluate reproducibility. Next, the sequencing reads were first subjected to quality control using FastQC v0.11.5 (29) to assess the quality of the raw data. Low-quality reads and adapter sequences were trimmed using Trimmomatic v0.39 (30). The resulting data were aligned to the Bactrian camel reference genome (Ca_bactrianus_MBC_1.0) using BWA v0.7.17 (31). Post-alignment, the alignment rate was calculated to assess sequencing efficiency and accuracy. The alignment files were processed using SAMtools v1.17 (21) to convert, sort, and index the aligned reads. Alignment quality was further assessed by examining metrics such as the percentage of mapped reads, coverage depth, and uniformity of coverage across the genome. To ensure the chip’s precision and reliability in genotyping, we meticulously analyzed the genotype concordance rate among the duplicate samples. This analysis is vital for validating the chip’s capability to produce consistent and reproducible results. Additionally, we quantified the detection rates of all test samples across various site coverage depths, providing a comprehensive evaluation of the chip’s performance under different genomic conditions.

### 2.6 Analysis of genetic diversity and population structure

To evaluate the performance of the SNP array in detecting population genetic diversity and structure, we genotyped and analyzed 398 DNA samples. VCFtools v0.1.11 (23) was employed to filter the SNPs and individuals. Specifically, individuals and SNPs with a detection rate lower than 95% were excluded. SNPs with minor allele frequencies lower than 0.05 were excluded. SNPs with significant deviation from Hardy–Weinberg equilibrium (*p* < 0.01) in any population were excluded. The genotyping data were extracted and converted into PLINK. bed and .fam formats, and then imported into PLINK v1.90 (22) and ADMIXTURE v1.3.0 (32) software for analysis. Using PLINK, PCA is conducted to identify major sources of genetic variation across the six breeding areas. ADMIXTURE is used to infer population structure and assign individuals to genetic clusters. It provides estimates of the proportion of an individual’s genome that originates from each of K ancestral populations. The tested *K* was set from 2 to 9.

### 2.7 Genomic prediction

The GBLUP model is used to calculate GEBV as follows:


y=1μ+Xβ+Zu+e

where y is the vector of corrected phenotypic values. These are the phenotypic measurements (e.g., milk yield, milk protein, milk lactose milk fat) that have been adjusted for fixed effects (e.g., breed, parity, lactation period). 1 is a vector of ones, which ensures that the overall mean ( μ ) is added to each observation, with 𝜇 being the overall mean of the phenotypic values across all individuals in the study. Xβ represents the fixed effects, where 𝑋 is the incidence matrix for these effects, and 𝛽 is the vector of fixed effects coefficients. 𝑍 is the incidence matrix that relates the additive genetic values (𝑢) to the phenotypic values (𝑦), with each row of 𝑍 corresponding to an individual and each column to a genetic effect. 𝑢 is the vector of additive genetic values (or genomic breeding values, GEBVs). These values represent the genetic contribution of each individual to the trait. It is assumed that 𝑢 ∼ *N*(0, *Gσ*_𝑢_^2^), where G is the relationship matrix built with the HIBLUP v1.4.0 software (33). This matrix represents the genetic relationships between individuals based on SNP marker data. *σ*_𝑢_^2^ represents the additive genetic variance. 𝑒 is the vector of random residual effects, representing the variation in the phenotypic values not explained by the model. It is assumed that 𝑒∼*N* (0, *Iσ*_𝑒_^2^), where *I* is an identity matrix and *σ*_𝑒_^2^ is the residual variance.

Cross-validation (CV) is usually used to obtain a reliable and stable model, and to evaluate the quality of the model (34). To evaluate the accuracy of genomic prediction, we utilized five-fold cross-validation. The dataset was divided into five approximately equal-sized groups. In each iteration of the cross-validation, four groups were used as the training set to estimate model parameters, while the remaining group was used as the validation set to test the model’s predictive performance. Prediction accuracy was calculated as the correlation between the predicted estimated breeding values (EBVs) and the actual phenotypes in the validation set, divided by the square root of the heritability estimated in the validation population. The standard error (SE) was calculated as the standard deviation of the five calculated reliability values from the five-fold cross-validation, divided by the square root of five.

## 3 Results

### 3.1 Variant calling from transcribed region sequences

A total of 19,177 confident SNPs were detected from the RNA-seq data of lactating Bactrian camels. Initially, transcriptomes of peripheral blood from 125 selectively chosen individuals, exhibiting consistent lactation period out of 1,243 Bactrian camels, were sequenced. This sequencing yielded approximately 3,575 million paired-end reads, averaging 29 million paired-end reads per individual sample. After quality control, the base effectiveness rate stood at 97.88%. The Q30 ratio surpassed 93.14%, and the GC content exceeded 52.08%. Remarkably, about 91.72% of the reads were accurately mapped to the Bactrian camel genome (Ca_bactrianus_MBC_1.0), with nearly 80.54% of the reads from each individual uniquely aligning with the camel genome. Detailed alignment information for each sample is tabulated in Supplementary Table S1. Subsequently, a comprehensive SNP calling was conducted in the reference genome from 125 transcriptome datasets, unveiling 9,790,170 SNPs. Only those SNP variants where the alternative allele appeared in all samples and had a Phred quality score of at least 25 and a minimum read depth of 10 were retained. Minor allele frequencies (MAFs) and missing rates were calculated, with SNPs having MAF <0.05 excluded. Additionally, we tested for Hardy–Weinberg equilibrium (HWE) using the -hwe option and removed SNPs that significantly deviated from HWE (*p* < 0.01). Among these, 19,177 SNPs were confidently ascertained across the entire cohort.

### 3.2 Identification of SNPs associated with milk traits

**Figure 2:**
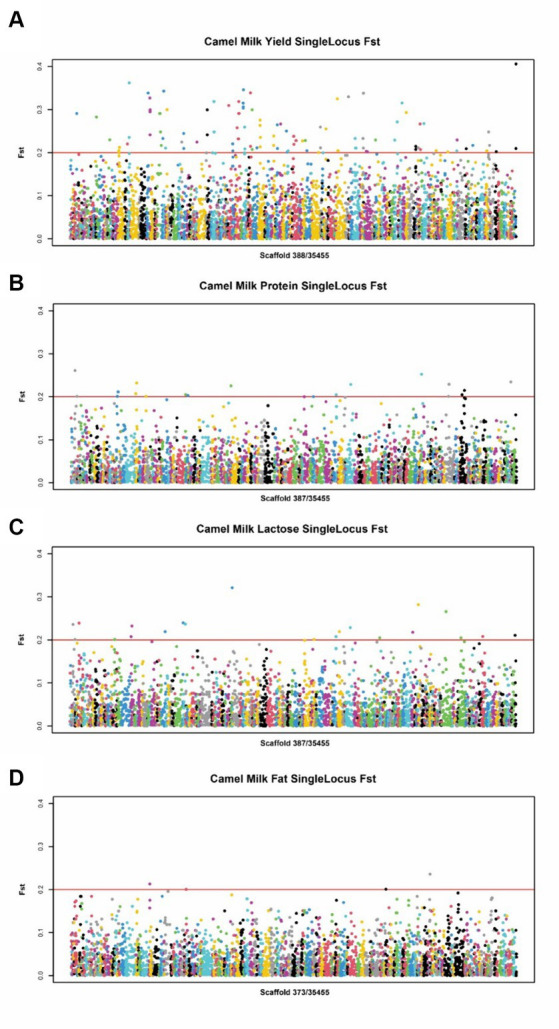
Manhattan plot of SNPs for milk traits by EWSS. **(A)** Manhattan plot of SNPs between high and low milk yield group. **(B)** Manhattan plot of SNPs between high and low milk protein group. **(C)** Manhattan plot of SNPs between high and low milk lactose group. **(D)** Manhattan plot of SNPs between high and low milk fat group.

**Figure 3:**
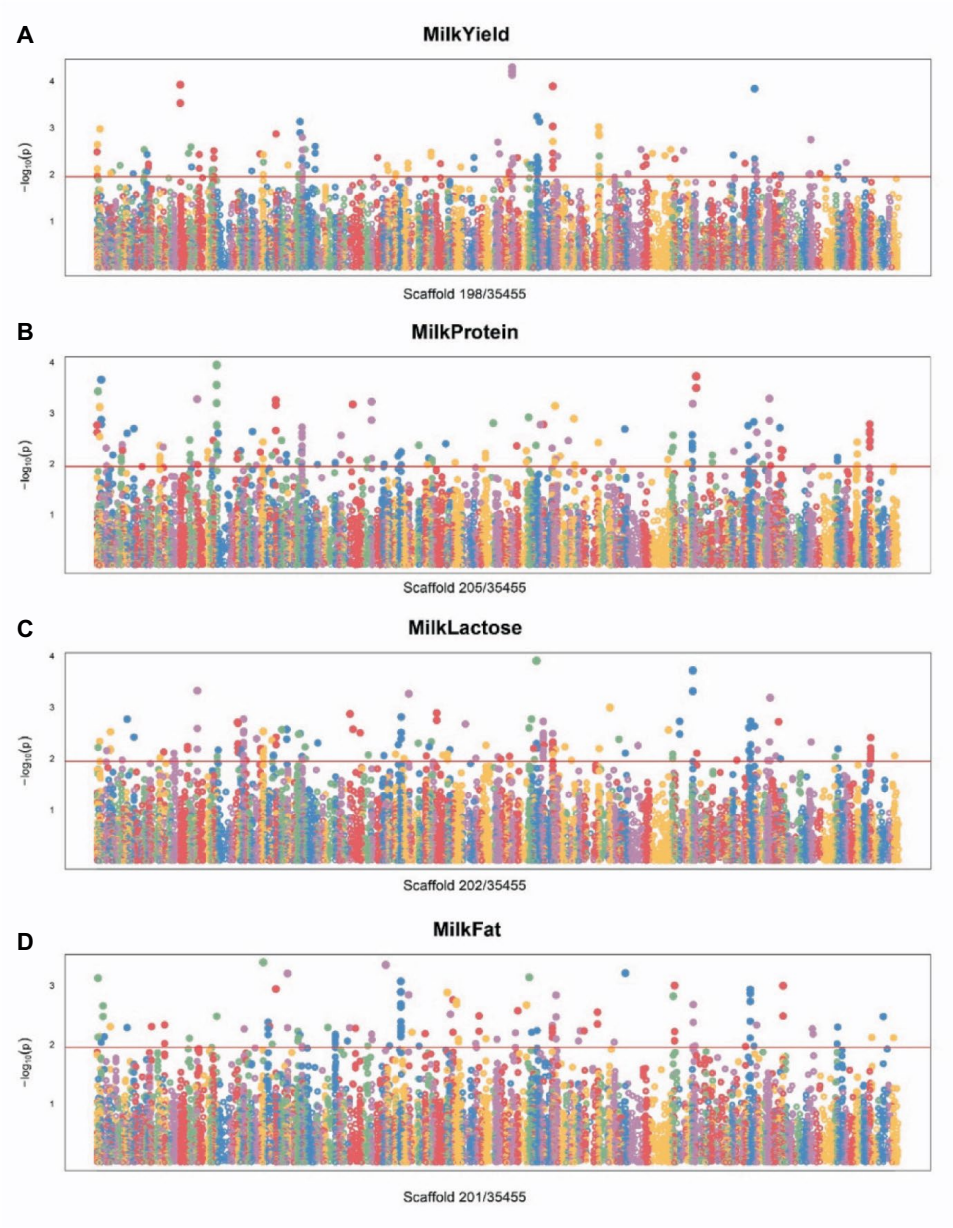
Manhattan plot of SNPs for milk traits by GWAS. **(A)** Manhattan plot of SNPs associated with milk yield traits. **(B)** Manhattan plot of SNPs associated with milk protein traits. **(C)** Manhattan plot of SNPs associated with milk lactose traits. **(D)** Manhattan plot of SNPs associated with milk fat traits.

**Figure 4:**
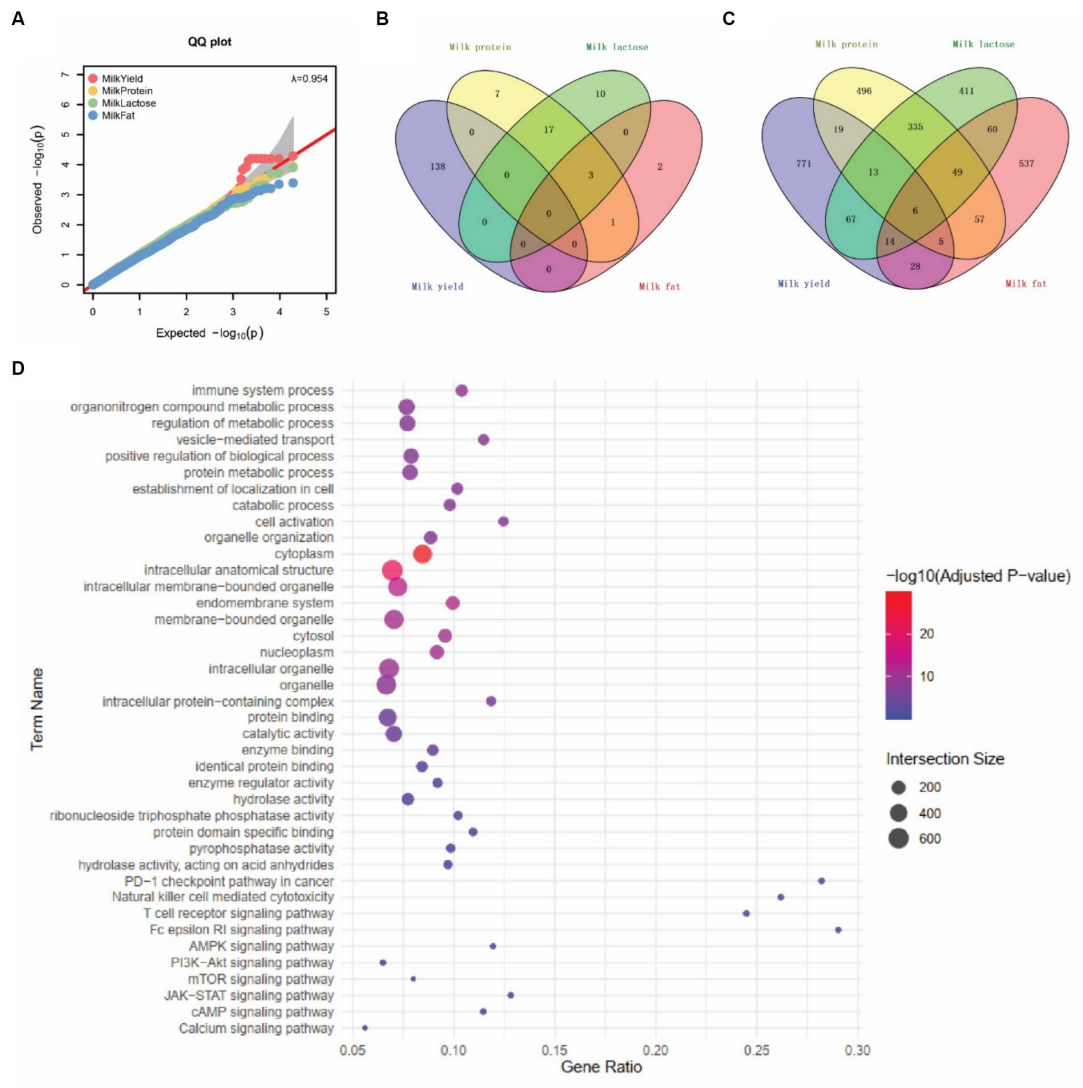
SNPs associated with four milk traits. **(A)** Quantile–quantile (QQ) plots represent the negative logarithms of the expected *p*-values (*x*-axis) and observed *p*-values (*y*-axis) (right panel). **(B)** SNPs associated with four milk traits by EWSS. **(C)** SNPs associated with four milk traits by GWAS. **(D)** Top 10 Gene Ontology (GO) and KEGG pathway terms associated with milk trait-related snp genes.

For the lactation performance traits of milk yield, milk protein, milk lactose, and milk fat, both Exon Wide Selection Signature (EWSS) and Genome Wide Association Study (GWAS) methodologies were employed to generate a comprehensive SNP set. Initially, the calculation of *F*_ST_ was applied to 19,177 SNPs to discern associations with extreme phenotypic milk traits. Groupings according to extreme values of milk traits are shown in Table 1. The statistical analysis of other milk components in each extreme value group is shown in Tables 2–5. Using a threshold of *F*_ST_ > 0.2, we identified 178 loci as outliers associated with various milk traits. Specifically, 138 loci were related to milk yield, 28 to milk protein, 30 to milk lactose, and 6 to milk fat. These loci are depicted in Figure 2. These identified loci serve as a partial reference for the selection of SNPs associated with lactation traits. Simultaneously, leveraging the 19,177 SNPs identified within the transcribed regions, we also employed GWAS to associate them with four continuous lactation performance traits across 125 subjects, thereby providing an additional reference set for the screening of pertinent loci. Therefore, significant associations were found with 923 SNPs for milk yield, 980 SNPs for milk protein, 955 SNPs for lactose, and 756 SNPs for milk fat traits (FDR <0.05), shown in Figure 3. During the analysis, we calculated the genomic inflation factor (λ) to assess the potential inflation of test statistics due to population structure or cryptic relatedness. The results showed a genomic inflation factor of 0.945, indicating minimal inflation and suggesting that the test statistics were not significantly affected by population structure or cryptic relatedness (Figure 4A). The overlap of SNPs associated with different traits is shown in Figures 4B,C. Overall, the amalgamation of both methods furnished us with a reference set comprising 2,960 SNP loci, which are associated with four lactation traits and dispersed within the transcribed regions of 1,395 genes, shown in Supplementary Table S2. A total of 81 overlapping sites were identified between *F*_ST_ and GWAS analyses. The functional enrichment analysis conducted on the identified genes indicated a significant overrepresentation in several biological processes (BP), particularly in areas related to the immune system, organonitrogen compound metabolism, and regulation of metabolic processes. Additional processes such as vesicle-mediated transport, positive regulation of biological processes, protein metabolism, and cellular localization establishment were also notably enriched. Furthermore, genes involved in catabolic processes were highlighted as part of the biological process category. To provide a comprehensive overview, the top 10 items from each category—Biological Processes (BP), Cellular Components (CC), Molecular Functions (MF), and Kyoto Encyclopedia of Genes and Genomes pathways (KEGG) were visually summarized in Figure 4D.

**Table 1:** The phenotype group of 4 milk traits.

Groups	Traits	Sample size	Mean	Min	Max	SD
HMY	High milk yield (kg)	15	2.28	2.05	2.70	0.10
LMY	Low milk yield (kg)	15	0.56	0.32	0.81	0.18
HMP	High milk protein (%)	15	4.11	4.01	4.26	0.08
LMP	Low milk protein (%)	15	2.82	2.25	3.33	0.29
HML	High milk lactose (%)	15	5.57	5.45	5.84	0.14
LML	Low milk lactose (%)	15	3.73	3.04	4.19	0.31
HMF	High milk fat (%)	15	8.38	7.53	10.00	0.84
LMF	Low milk fat (%)	15	2.49	1.04	3.58	0.95

**Table 2:** Statistical analysis of milk components in extreme milk yield group.

Milk component	High milk yield group	Low milk yield group	*p*-value
Milk yield, kg/day	2.28 ± 0.10	0.56 ± 0.18	<0.01
Milk protein, %	3.67 ± 0.06	3.7 ± 0.15	0.79
Milk lactose, %	5.05 ± 0.14	5.06 ± 0.32	0.95
Milk fat, %	5.80 ± 0.58	5.96 ± 0.33	0.56

**Table 3:** Statistical analysis of milk components in extreme milk protein group.

Milk component	High milk protein group	Low milk protein group	*p*-value
Milk yield, kg/day	1.08 ± 0.64	1.16 ± 0.0.83	0.75
Milk protein, %	4.11 ± 0.08	2.82 ± 0.29	<0.01
Milk lactose, %	5.53 ± 0.02	4.59 ± 0.21	<0.01
Milk fat, %	5.07 ± 0.27	4.76 ± 0.23	0.25

**Table 4:** Statistical analysis of milk components in extreme milk lactose group.

Milk component	High milk lactose group	Low milk lactose group	*p*-value
Milk yield, kg/day	1.29 ± 0.40	1.44 ± 0.47	0.49
Milk protein, %	4.03 ± 0.01	2.96 ± 0.12	<0.01
Milk lactose, %	5.57 ± 0.14	3.73 ± 0.31	<0.01
Milk fat, %	5.02 ± 1.77	4.96 ± 0.80	0.83

**Table 5:** Statistical analysis of milk components in extreme milk fat group.

Milk component	High milk yield group	Low milk yield group	*p*-value
Milk yield, kg/day	1.12 ± 0.20	1.41 ± 0.46	0.12
Milk protein, %	3.98 ± 0.02	3.51 ± 0.14	0.64
Milk lactose, %	5.44 ± 0.03	4.83 ± 0.31	0.37
Milk fat, %	8.38 ± 0.84	2.49 ± 0.95	<0.01

Values are presented as mean ± standard deviation (SD).

### 3.3 SNPs for the “CamelBell No. 1” array

**Figure 5:**
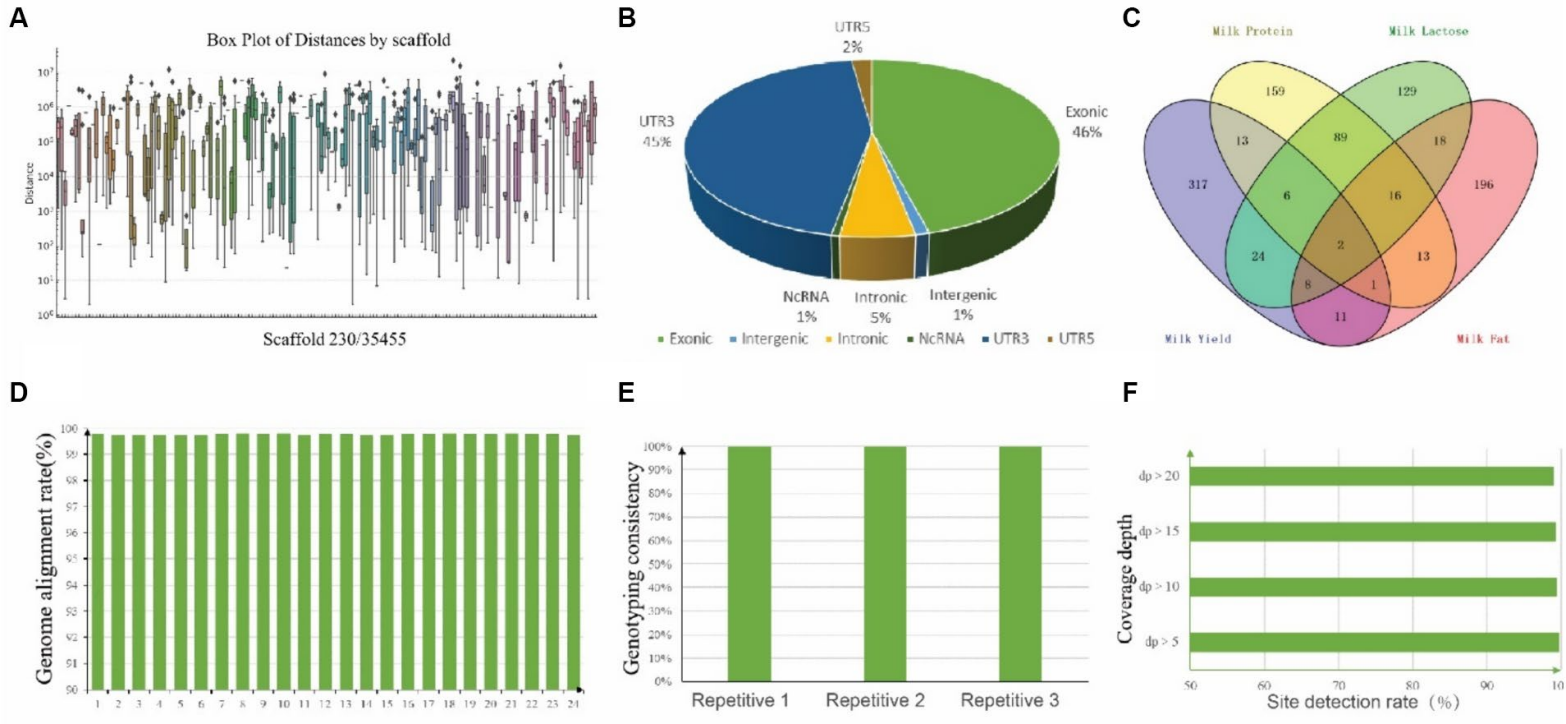
Annotation of 1,002 SNPs and Quality assessment of CamelBell No. 1 SNP array. **(A)** Box plot of SNP distances by 230 scaffolds. The *x*-axis lists the chromosomes, and the *y*-axis represents the distance between adjacent SNPs on a logarithmic scale. **(B)** Pie chart depicting the distribution of SNP positions across the genome. **(C)** Venn diagram illustrating the overlap of SNPs associated with various lactation traits. **(D)** Genome alignment rates achieved using the CamelBell No. 1 SNP array. **(E)** Genotyping consistency of duplicate samples analyzed with the CamelBell No. 1 SNP Array. **(F)** Site detection rates at varying coverage depths using the CamelBell No. 1 SNP array.

To explore the variation in SNP density across different chromosomes, a box plot was generated (Figure 5A). The median distances and the ranges vary significantly, with some scaffold showing a higher concentration of closely spaced SNPs. To mitigate the incidence of false positives in genotyping, SNPs were judiciously selected for inclusion on the chip, guided by criteria such as SNP quality, polymorphism status, SNP density, and the extent of association with specific traits. In brief, we submitted 2,960 previously identified candidate SNPs associated with lactation traits to the Targeted Capture Sequencing Probe Design System, and 2,681 SNPs passed evaluation. Next, considering the location and function of these SNPs, we eliminated one of the two sites within less than 100 bp from each other, focusing on retaining sites supported by existing literature. Thus, 1,002 SNPs were finally manually selected for the “CamelBell No. 1” array. For those SNP site, there were 460 SNPs located in exon region, 54 located in intron region and 10 located in intergenic region, shown in Figure 5B, and the details are in Supplementary Table S3. The association of these SNPs with lactation traits is visually represented in Figure 5C, with 201 SNPs being correlated with two or more lactation traits.

### 3.4 Genotyping performance of SNP array

The efficacy of the SNP array was assessed through the genotyping of 24 DNA samples from Alashan League, Inner Mongolia. Inclusion of three pairs of replicate samples facilitated the evaluation of the chip’s detection performance. A genome alignment rate exceeding 99% was achieved for all samples, culminating in an average alignment rate of 99.77%, shown in Figure 5D. Separate testing of three duplicate samples yielded a typing consistency of 100% for each pair, underscoring the chip’s detection stability (Figure 5E). Comprehensive statistics pertaining to the detection rates across all test samples revealed that with a target site coverage depth exceeding 5×, an average site detection rate of 99.72% was maintained. Remarkably, even at a coverage depth surpassing 20×, the detection rate remained robust at 99.07%, shown in Figure 5F.

### 3.5 Genetic diversity of the core breeding populations

**Figure 6:**
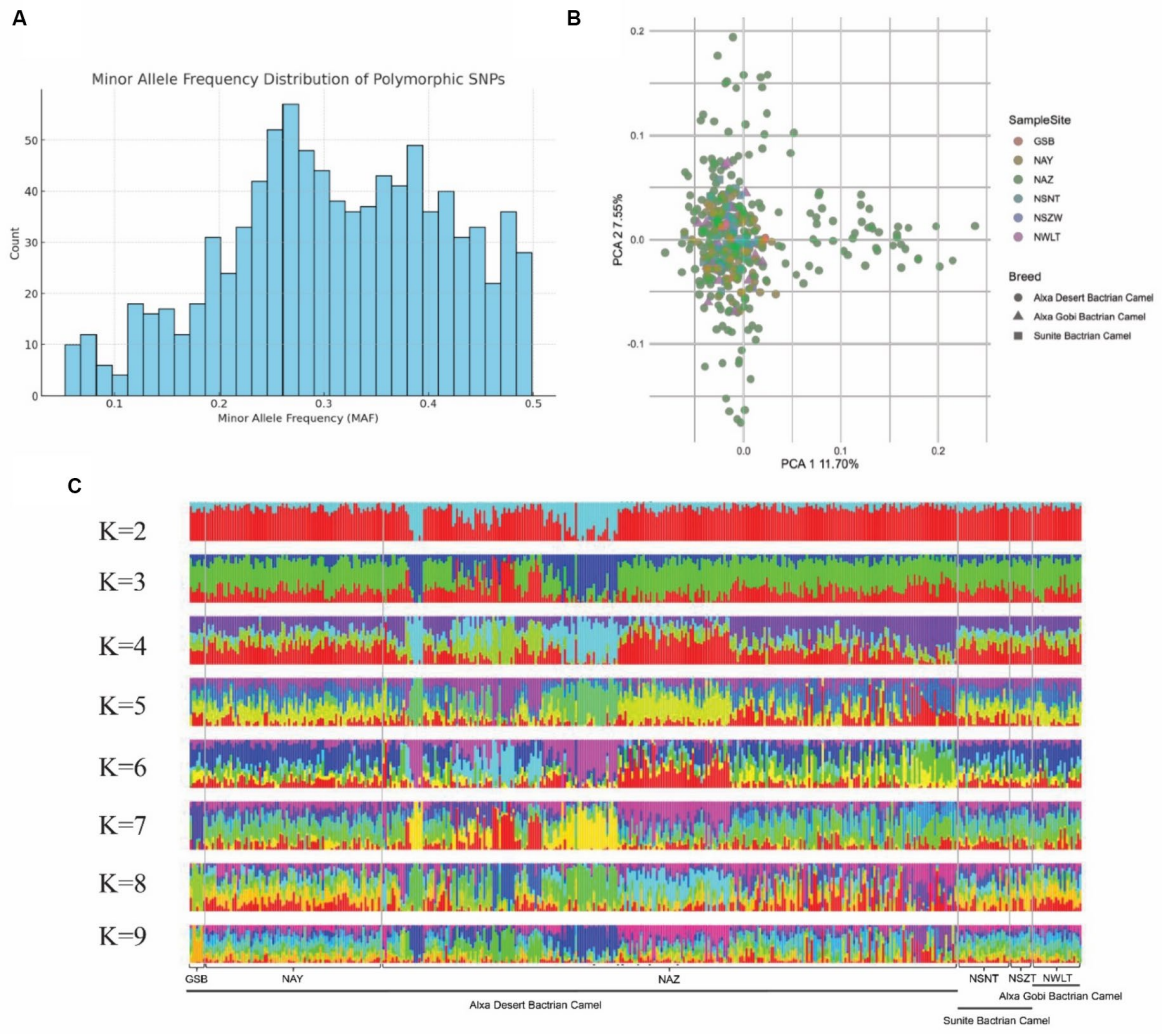
Genetic structure of camel core breeding population. **(A)** Bar plot of minor allele frequency (MAF) distribution. **(B)** PCA plot of the first and second PCs showing genetic distances among samples. Samples from different locations are represented in different colors. **(C)** Admixture plot (*K* = 2–9) for the 398 large camel individuals. Each individual is shown as vertical bar divided into *K* colors.

To assess the genetic diversity of the core breeding populations in of dairy Bactrian camels in northwest China, we collected 398 camels from six representative breeding areas, and conducted SNP genotyping using the SNP array. Firstly, we calculated the minor allele frequency (MAF) distribution of the population and plotted the histogram, as shown in Figure 6A. The MAF data from SNP array genotyping revealed that 95% of SNPs had a minor allele frequency greater than 0.1. Subsequently, we estimated the genetic structure and distance, observing minimal genetic divergence among samples from different breeding areas (Figures 6B,C). These results indicate that camels from six breeding areas share a similar genetic background regarding lactation functionality.

### 3.6 The prediction accuracy of CamelBell No. 1 SNP array

**Figure 7:**
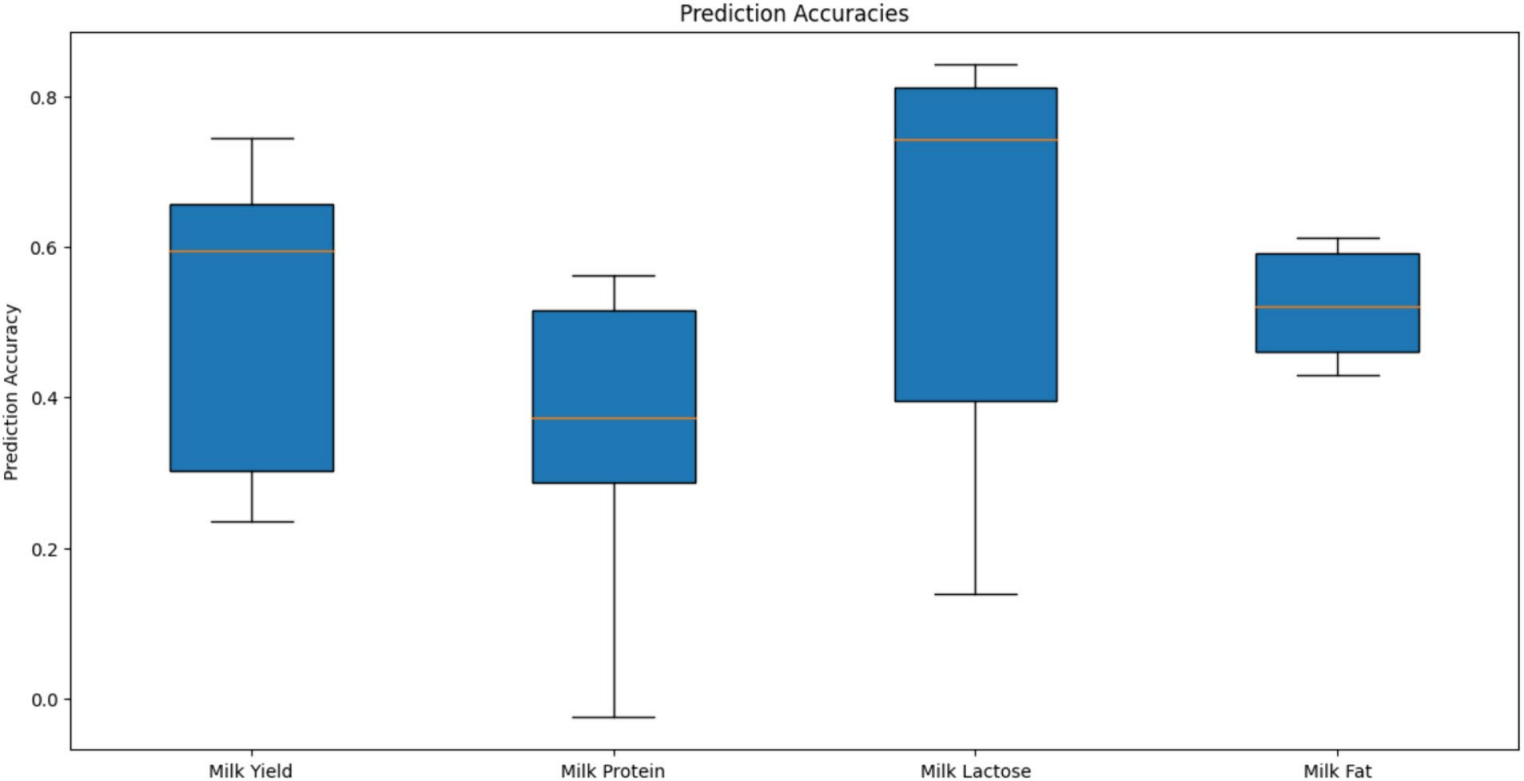
Prediction accuracies of genomic estimated breeding values for different milk traits using GBLUP in camel.

Genomic prediction (GBLUP) accuracy for the four milk traits was assessed using genotype information from 914 markers that passed the quality control filters (MAF >0.01, Call Rate >0.9). A total of 398 camels were randomly divided into training (80%) and validation (20%) sets for cross-validation, and this process was repeated five times to ensure robust evaluation. The box plot of prediction accuracy values is shown in Figure 7. The genomic prediction accuracies for the four milk traits vary, with milk lactose showing the highest and most consistent accuracy, followed by milk yield and milk fat, while milk protein exhibits the lowest and most variable accuracy. The mean Pearson correlation coefficients of the four milk traits were 0.27, 0.30, 0.34, and 0.44, respectively, indicating modest linear relationships between the predicted Genomic Estimated Breeding Values (GEBVs) and the actual phenotypes. Among these traits, milk fat exhibited the strongest correlation. The standard error (SE) values of the estimated correlation coefficients for all traits were less than 0.10. The mean regression coefficients for the four milk traits were 0.88, 0.76, 1.14, and 0.69, respectively. These values suggest that while predictions for milk yield are relatively unbiased, predictions for milk protein and milkfat tend to underestimate the actual genetic values, and predictions for milk lactose tend to overestimate them. The SE values of the estimated regression coefficients for all traits were less than 0.06.

## 4 Discussion

In this study, we developed a new SNP array (CamelBell No. 1) in Bactrian camel based on genotyping by target sequencing. To our knowledge, this is the inaugural liquid chip specifically designed for dairy Bactrian camel breeding. After collecting lactation performance and phenotype data from thousands of lactating Bactrian camels in Northwest China, we handpicked 523 to establish a core breeding group. From this pool, we further selected 125 camels that shared the same peak lactation period and identical feeding conditions, aiming to identify single nucleotide polymorphisms (SNPs) linked to lactation traits. Utilizing these 1,002 SNPs, we advanced the genetic enhancement of dairy Bactrian camel breeding. When SNPs are identified using RNA-seq data, a limited number of individuals can effectively pinpoint loci exhibiting significant polymorphism (35). Given that lactation traits are quantitative and influenced by a multitude of genes and environmental factors, a larger sample size is instrumental in deciphering the genetic architecture of these complex traits. Consequently, in this study, RNA-seq data from the 125 lactating Bactrian camels were chosen as the reference dataset for SNP discovery, providing a comprehensive basis for understanding the genetic determinants of lactation characteristics. One advantage of SNP discovery using RNA-seq is the versatility of the sequenced data, which extends beyond initial research objectives. This data can be repurposed for further investigative queries, including the exploration of how organisms adapt to varying environmental conditions. This multifaceted utility enhances the value of RNA-seq in genetic research (36). This method, widely adopted in genomic research, has proven effective in identifying SNPs associated with traits such as muscle yield and quality in fish (37), as well as thermo-resistant in oysters (38). Additionally, it has been instrumental in unraveling the genetic mechanisms behind tail fat deposition in sheep (9) and lactation performance in dairy cows (7), highlighting its versatility and importance in the field of animal genetics.

In our research, we employed Extended Window Sum Statistic (EWSS) to calculate fixation indices (*F*_ST_) (39, 40) for populations exhibiting significant variations in lactation performance. This approach allows us to screen for SNP variants in extreme phenotypic groups after controlling for environmental variables. Concurrently, we adopted the principles and methodologies of Genome-Wide Association Studies (GWAS) (41, 42) to investigate the correlation between lactation performance and polymorphic sites in transcription regions of the Bactrian camel. By integrating these two methods, we were able to screen for the most pertinent functional loci in Bactrian camels, providing a foundational set of functional loci crucial for chip design. However, despite our efforts to control for various confounding factors in our analysis, several potential confounding factors may still influence our results. These include environmental variation, physiological and health status, and genetic background. Specifically, although we standardized diet and housing conditions, other environmental factors such as microclimatic conditions, handling practices, and feed intake were not explicitly controlled. Variations in the health status or physiological conditions of the camels, such as subclinical infections, stress levels, and hormonal imbalances, were not measured or controlled. While principal component analysis (PCA) was used to account for major population structure, subtle genetic stratification or cryptic relatedness within the population may still exist. These unaccounted confounding factors may introduce biases or residual confounding in our GWAS results, potentially affecting the generalizability and validity of our findings. Future research should aim to measure and control for these variables more comprehensively to ensure more accurate and reliable genetic association results.

Compared to traditional silicon-based SNP panels, Genotyping By Targeted Sequencing (GBTS) (43) panels offer enhanced flexibility in handling varying sample sizes for genotyping. The GBTS marker system offers the flexibility to create multiple marker panels from a single master panel. It allows researchers to select a specific number of markers tailored to their unique research goals. While the breeding of dairy camels, including *Camelus dromedarius* and *Camelus bactrianus*, is progressing in various countries (44–46), conventional breeding methods still predominate. This study lies in its contribution to the breeding of camels with desirable milk production traits, such as milk yield, protein content, fat content, and lactose content, bolstered by advancements in Bactrian camel genome research. The development of CamellBell No. 1 provides technical support for the large-scale promotion of dairy breeding in Bactrian camel.

Our evaluation of the array’s typing performance revealed high levels of consistency and stability. Remarkably, even at a coverage depth exceeding 20×, the detection rate remained robust at 99.07%. This reliability is crucial for the chip’s application in further genetic studies. We used the chip to sequence DNA from individuals in core breeding groups across six regions to analyze their population structure. The analysis of 398 individuals did not show complete separation in terms of lactation function, suggesting a common ancestral origin or an early stage in the selection process. The breeding values were estimated using the GBLUP model, and the accuracy of trait prediction was evaluated using five-fold cross-validation. We found that although the model for the lactose trait had the risk of overfitting, its prediction accuracy was the highest. This high accuracy may be attributed to the genetic stability of the lactose trait. Lactose percent has been reported to be highly heritable (0.53), according to a study in Holstein cows from Michigan (47). The statistical model, marker density, and sample size influenced on selection accuracy (48). Due to the low samples size in this research, there may be bias in the prediction. Additional animals are required to fully evaluate the array for genomic selection (GS), and we are working to increase the sample sizes of the reference and candidate populations for genotyping with the SNP array. Given that large-scale breeding of Bactrian camels is not as established as in cows and other animals, and considering the challenges in sampling, low-cost and highly flexible liquid phase chips like CamelBell No. 1 play a vital role in accelerating the breeding of dairy Bactrian camels.

## 5 Conclusion

In summary, this study is the first try to report a functional liquid chip of Bactrian camel, CamelBell No. 1 SNP array, and conduct a comprehensive evaluation of the chip’s typing performance, with a view to providing tools and carriers for dairy Bactrian camels breeding.

## Data availability statement

The data presented in the study are deposited in the SRA repository, accession number PRJNA1127254.

## Ethics statement

The animal studies were approved by Inner Mongolia Agricultural University Laboratory Animal Welfare and Ethics Committee. The studies were conducted in accordance with the local legislation and institutional requirements. Written informed consent was obtained from the owners for the participation of their animals in this study.

## Author contributions

LG: Methodology, Visualization, Writing – original draft. LD: Writing – review & editing, Writing – original draft. BL: Software, Validation, Writing – review & editing. JW: Validation, Writing – review & editing. ZL: Formal analysis, Writing – review & editing. FM: Investigation, Writing – review & editing. BM: Data curation, Writing – review & editing. CC: Investigation, Writing – review & editing. YB: Data curation, Writing – review & editing. YG: Data curation, Writing – original draft. CS: Supervision, Writing – original draft. JC: Conceptualization, Project administration, Writing – review & editing. WZ: Conceptualization, Funding acquisition, Project administration, Resources, Writing – review & editing.

## Footnotes

^1^ https://www.ncbi.nlm.nih.gov/datasets/taxonomy/9837/

^2^ https://biit.cs.ut.ee/gprofiler/gost
