# Supplementary material for: Development and application of a 1K functional liquid chip for lactation performance in Bactrian camels
Source: Front Vet Sci. 2024 Jul 2;11:1359923. doi: 10.3389/fvets.2024.1359923 (PMC11253134; doi:10.3389/fvets.2024.1359923)
Supplement: Supplementary file 4 [file Data_Sheet_1.PDF]

RNA-Seq pipeline script

```
#################### QUALITY CONTROL ####################

INPUT=/home/camel/clean_data/
find $INPUT -name "*_1.clean.fq.gz"  |  sed 's/_1.clean.fq.gz$//' | xargs -n 1 -P 15 -I PREFIX \
    sh -c '
fastq=`echo PREFIX |sed "s/\(^.*\/\)\(.*\)/\2/" `
if [ ! -f PREFIX.html ];then
/home/Biosoftware/fastp -i PREFIX_1.clean.fq.gz -o PREFIX_filter_1.fastq.gz \
       -I PREFIX_2.clean.fq.gz -O PREFIX_filter_2.fastq.gz \
       -Q --thread=2 --length_required=50 --n_base_limit=6 -q 20 -u 60 --compression=6 -c -h PREFIX.html -R PREFIX
fi
    '
#################### INDEX ####################
#!/bin/bash

ref=/home/camel/camel-ref
fasta=$ref/GCF_000767855.1_Ca_bactrianus_MBC_1.0_genomic.fna
index=$ref/Ca_bactrianus_MBC_1.0
gtf=$ref/GCF_000767855.1_Ca_bactrianus_MBC_1.0_genomic.gff

hisat2_extract_exons.py $gtf> $ref/Ca_bactrianus.exon
hisat2_extract_splice_sites.py $gtf> $ref/Ca_bactrianus.ss

hisat2-build -p 28 $fasta --ss $ref/Ca_bactrianus.ss --exon $ref/Ca_bactrianus.exon $index

#################### ALIGNMENT ####################

#!/bin/bash
INPUT=/home/camel/clean_data
index=/home/camel/camel-ref/Ca_bactrianus_MBC_1.0
OUTPUT=/home/camel/clean_data/bam

find $INPUT -name "*filter_1.fastq.gz"  |  sed 's/_filter_1.fastq.gz$//' | xargs -n 1 -P 8 -I PREFIX     sh -c '
fastq=`echo PREFIX |sed "s/\(^.*\/\)\(.*\)/\2/" `

if [ -e PREFIX.html ];then
hisat2 -p 3 --dta -x '$index' -1 PREFIX_filter_1.fastq.gz -2 PREFIX_filter_2.fastq.gz -S
'$OUTPUT'/align/$fastq.aligned.sam 2>'$OUTPUT'/align/$fastq.aligned.log
samtools sort -@ 3 -o '$OUTPUT'/align/$fastq.aligned.bam '$OUTPUT'/align/$fastq.aligned.sam
rm -rf '$OUTPUT'/align/$fastq.aligned.sam

#################### REMOVE DUPLICATES AND  VARIANT CALLING####################

find /home/camel/clean_data/bam/ -type f -name "*.bam" >bam.list

/home/Biosoftware/samtools mpileup -BIg -q 20 -d 1000 -f /home/camel/camel-ref/Ca_bactrianus_MBC_1.0.genome.fa -b
bam.list | bcftools call -mv -V indels  -o camelRNA-seq.vcf
```
